# Interactions between the flavescence dorée phytoplasma and its insect vector indicate lectin-type adhesion mediated by the adhesin VmpA

**DOI:** 10.1038/s41598-021-90809-z

**Published:** 2021-05-27

**Authors:** Nathalie Arricau-Bouvery, Sybille Duret, Marie-Pierre Dubrana, Delphine Desqué, Sandrine Eveillard, Lysiane Brocard, Sylvie Malembic-Maher, Xavier Foissac

**Affiliations:** 1 Univ. Bordeaux, INRAE, Biologie du Fruit et Pathologie, UMR 1332, 33140 Bordeaux, Villenave d’Ornon France; 2 Univ. Bordeaux, CNRS, INSERM, Bordeaux Imaging Center, BIC, UMS 3420, US 4, 33140 Bordeaux, Villenave d’Ornon France

**Keywords:** Bacteria, Cellular microbiology

## Abstract

The flavescence dorée phytoplasma undergoes a propagative cycle in its insect vectors by first interacting with the insect cell surfaces, primarily in the midgut lumen and subsequently in the salivary glands. Adhesion of flavescence dorée phytoplasma to insect cells is mediated by the adhesin VmpA. We hypothesize that VmpA may have lectin-like activity, similar to several adhesins of bacteria that invade the insect gut. We first demonstrated that the luminal surface of the midgut and the basal surface of the salivary gland cells of the natural vector *Scaphoideus titanus* and those of the experimental vector *Euscelidius variegatus* were differentially glycosylated. Using ELISA, inhibition and competitive adhesion assays, and protein overlay assays in the Euva-6 insect cell line, we showed that the protein VmpA binds insect proteins in a lectin-like manner. In conclusion, the results of this study indicate that *N*-acetylglucosamine and mannose present on the surfaces of the midgut and salivary glands serve as recognition sites for the phytoplasma adhesin VmpA.

## Introduction

Diseases caused by phytoplasmas affect more than 1000 plant species, resulting in severe symptoms and notable economic losses in agricultural crops^1–4^. For example, controlling flavescence dorée, a quarantine disease-impacting European grapevine, is associated with economic and environmental costs due to compulsory surveys and insecticide treatments, removal of infected plants and production losses^5–7^. Phytoplasmas are small cell wall-less bacteria and have been classified into 43 “*Candidatus* Phytoplasma” species to date^8^. In plants, phytoplasmas are restricted to phloem sieve elements and are naturally transmitted from plant to plant by phloem sap-sucking insects belonging to the order Hemiptera, specifically Cicadellidae (leafhoppers), Fulgoroidea (planthoppers) and Psylloidea (psyllids)^9,10^. Elucidating the interactions between phytoplasmas and insect vectors at the molecular level is highly important to reduce insecticide treatments and develop innovative control strategies. Phytoplasmas are transmitted in a persistent manner and undergo a propagative and multiplicative cycle into their insect host with colonization of the midgut and salivary glands^11^. The duration of the acquisition, latency and inoculation access periods depends on the phytoplasma/insect vector couples^12–15^. The spatiotemporal dynamics of the ‘*Ca.* P. asteris’-related strain onion yellows (OY) phytoplasma in the insect vector *Macrosteles striifrons* were precisely described by Koinuma et al.^16^. The study by these researchers showed that the first organ to be colonized was the anterior midgut between 7 and 14 days after acquisition began. The salivary glands, particularly the type III cells, were later colonized by the phytoplasmas that multiplied starting from 21 days after acquisition. Thus, the life cycle of phytoplasmas in their insect vectors implies invasion of diverse cells. Interactions between phytoplasma surface-exposed proteins and insect proteins are necessary to enable the adhesion of phytoplasmas to the apical surface of gut cells. This adhesion constitutes the initial and essential step of insect vector colonization. Adhesion to salivary gland cells is also an important step for phytoplasmas to colonize these cells and reach the saliva to be transmitted to their host plants. The first example of the interaction of phytoplasma surface-exposed proteins with insect proteins was described with OY and chrysanthemum yellows (CY) phytoplasmas. The antigenic membrane protein (Amp) interacts with microfilaments, actin, and ATP synthase of the insect vectors *M*. *striifrons*, *Macrosteles quadripunctulatus* and *Euscelidius variegatus*^17–19^. These interactions contribute to insect transmissibility, probably enabling phytoplasma movement within insect cells. Several other surface-exposed proteins were found in the genome of phytoplasmas and are supposed to be implicated in cell invasion by interacting with insect proteins. This was hypothesized for P38 by demonstrating its interaction with insect proteins in vitro^20^, and for the immunodominant membrane proteins Imp and IdpA and the variable membrane proteins Vmps, partly because, as a measure for Amp, they are subjected to strong selective pressure^21–25^.

Propagation of flavescence dorée phytoplasmas (FDPs) is naturally achieved in vineyards by the leafhopper vector *Scaphoideus titanus*^26^. Both nymphs and adults are able to transmit FDPs to grapevines or broad beans used for experimental transmissions^27–31^. FDP can also be transmitted by *Euscelidius variegatus* from fava bean to fava bean, where they multiply^32^. The FDP surface-exposed proteins Imp and VmpA were observed to interact with insect proteins that have not been identified to date^33,34^. VmpA is implicated in FDP adhesion to insect cells in culture and to the perimicrovillar membrane that covers the microvilli of midgut epithelial cells of *E*. *variegatus*^33^. Moreover, VmpA is expressed by phytoplasmas invading salivary glands^33^ and is also believed to play a role in adhesion to salivary gland cells. In insects, various glycoconjugates are exposed to the surface of the perimicrovillar membrane and the basal surface of salivary gland cells; these glycoconjugates include mannose, glucose, galactose, *N*-acetylglucosamine, *N*-acetylgalactosamine and 5-*N*-acetylneuraminic acid^35–40^. Many adhesins of bacteria and parasites bind the glycoconjugates of the gut of their insect vectors; these adhesins include HxfA and HxfB of *Xylella fastidiosa*^41,42^. We hypothesized that VmpA may act as a lectin binding to the sugar moiety of surface glycoproteins of insect vector cells. Using fluorescent lectins and microscopic observations, we demonstrated that the luminal surface of the midgut and basal surface of the salivary glands of *S*. *titanus* and *E*. *variegatus* are glycosylated. ELISA, inhibition and competitive adhesion assays, and protein overlay assays were used to show that VmpA attaches to insect vector cells in a lectin-like manner.

## Results

### Presence of similar glycoconjugate patterns at the surface of midgut and salivary gland cells of *E*. *variegatus* and *S. titanus*

**Figure 1:**
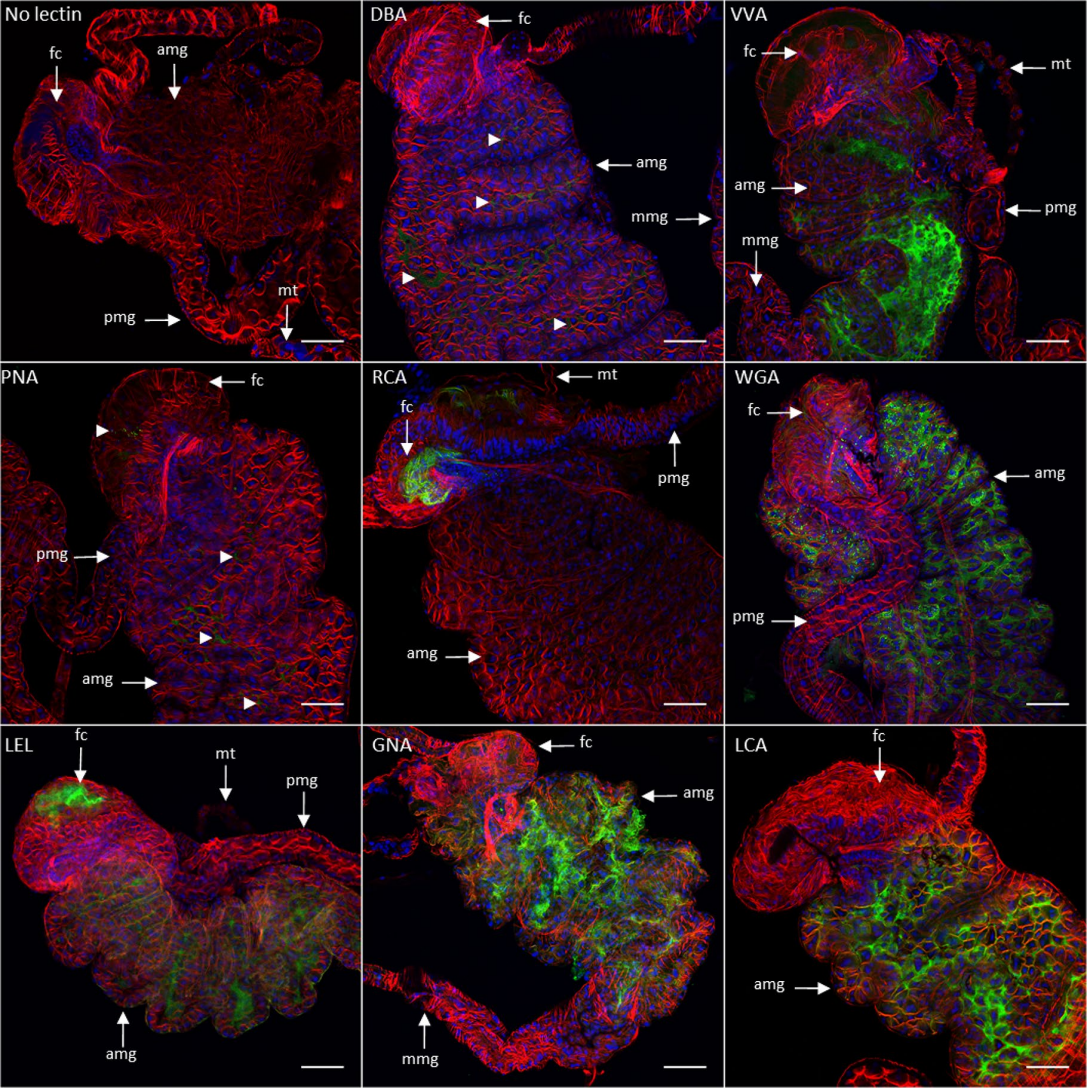
Observation of ingested fluorescent lectins binding midgut carbohydrates of *E*. *variegatus* by laser scanning microscopy. Cell nuclei were stained with DAPI (blue), and actin filaments were stained with Alexa 568-phalloidin (red). The FITC-lectins were colored in green. The fluorescent lectins used to stain the cells are indicated in each picture except for the control, where fluorescent lectin was omitted. *amg* anterior midgut, *mmg* middle midgut, *pmg* posterior midgut, *fc* filter chamber, *mt* Malpighian tubules. Arrowheads in panels DBA and PNA indicate the presence of fluorescence. Scale bar 100 µm.

To determine which carbohydrates are present at the surfaces of the two main barriers that phytoplasmas must cross, that is, the luminal surface of midgut epithelial cells and the basal surface of salivary gland cells, we determined the binding capacity to these organs exhibited by fluorescent lectins with different specificities. The lectins utilized in this study, as listed in Table 1, were DBA- and VVA-binding glycoproteins containing *N*-acetylgalactosamine (GalNAc), PNA- and RCA-binding glycoproteins with β1,4- and β1,3-galactose, respectively, WGA- and LEL-binding glycoproteins with *N*-acetylglucosamine (GlcNAc), GNA-binding glycoproteins containing mannose (Man), and LCA-binding glycoproteins containing Man or glucose (Glc). After *E*. *variegatus* ingested the fluorescent lectins, their retention in the midgut lumen showed that the apical surface of the midgut was glycosylated (Fig. 1). A very weak fluorescent signal was observed along the gut lumen when fluorescent DBA and PNA lectins were used. The RCA lectin bound to glycoconjugates present in the filter chamber or close to it, but no fluorescence was detected in the midgut of *E*. *variegatus*. Stronger and more extensive labeling of the midgut was observed when the insects ingested VVA, WGA, LEL, GNA and LCA lectins, often including the filter chamber. Very similar results were obtained when midguts of *S*. *titanus* were observed using fluorescence microscopy after ingestion of these various lectins (see Supplementary Fig. S1 online).

**Table 1:** List of the lectins used with their primary sugar specificity.

Lectin name	Abbreviation	Primary sugar specificity
*Dolichos biflorus* agglutinin	DBA	αGalNAc
*Vicia villosa* agglutinin	VVA	α,βGalNAc
Peanut agglutinin	PNA	Galβ3GalNAc
*Ricinus communis* agglutinin	RCA I	βGal
Wheat germ agglutinin	WGA	α,βGlcNAc
*Lycopersicon esculentum* lectin	LEL	(GlcNAc)_2–4_
Galanthus Nivalis agglutinin	GNA	α-1,3Man
*Lens culinaris* agglutinin	LCA	αMan, αGlc

*Gal* d-Galactose, *GalNAc* *N*-Acetylgalactosamine, *Glc* d-Glucose, *GlcNAc* *N*-Acetylglucosamine, *Man* Mannose.

**Figure 2:**
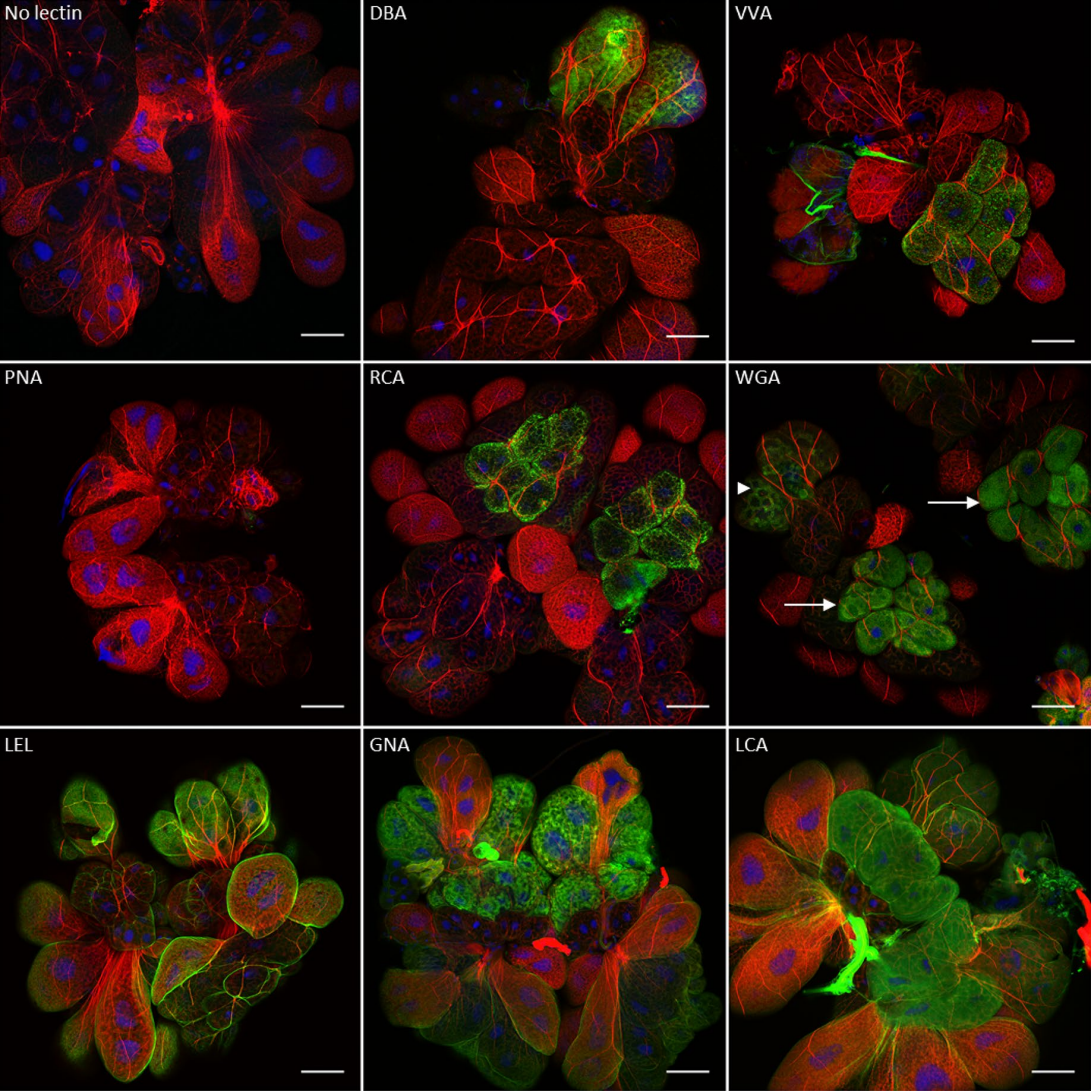
Observation of fluorescent lectins binding salivary gland cell carbohydrates of *E*. *variegatus* by laser scanning microscopy. Cell nuclei were stained with DAPI (blue), and actin filaments were stained with Alexa 568-phalloidin (red). The FITC-lectins were colored in green. The fluorescent lectins used to stain the cells are indicated in each picture except for the control, where fluorescent lectin was omitted. The arrowhead indicates one of the two cell types that possess glycoconjugates bound by the WGA lectin, and the arrows indicate the other cell type. Scale bar 100 µm.

The salivary glands of both insect vectors were dissected and incubated with the same fluorescent lectins. The results show that the salivary gland cells of *E*. *variegatus* are differentially labeled according to the cell type and lectin (Fig. 2). The lectin PNA bound the salivary gland cells very poorly. The DBA, VVA and RCA lectins bound only one cell type that was different depending on the lectin and could be differentiated by their size and actin network. The WGA lectin primarily fixed only two cell types that were distinguished by their size and labeling with the fluorescent lectin. This labeling was uniform at the surface of the first cell type, as indicated by arrows, and was restricted to structures in a network on the second cell type, as indicated by arrowhead. Finally, the LEL, GNA and LCA lectins fixed the surface of all the salivary glands somewhat abundantly, depending on the cell type. Similar results were obtained with the salivary glands of *S*. *titanus* (see Supplementary Fig. S2 online), except that the DBA lectin did not label any cell, and the PNA lectin was fixed to one cell type.

These results are summarized in Table 2. The two primary main differences between *E*. *variegatus* and *S*. *titanus* were the labeling of *E*. *variegatus* midgut and salivary glands with the DBA lectin, which was not observed in *S*. *titanus*, and the labeling of *S*. *titanus* salivary glands with the PNA lectin, which was not observed in *E*. *variegatus*.

**Table 2:** Binding of the fluorescent lectins to the apical surface of midgut and basal surface of salivary gland cells of *S*. *titanus* and *E*. *variegatus*.

Lectin	*E*. *variegatus* midgut		*S*. *titanus* midgut		*E*. *variegatus* salivary glands			*S*. *titanus* salivary glands		
	Non-infected		Non-infected		Non-infected	Infected		Non-infected	Infected	
	Intensity of fluorescence	Localization	Intensity of fluorescence	Localization	Number of cell types labelled	Number of cell types labelled	Presence of lectin and phytoplasmas	Number of cell types labelled	Number of cell types labelled	Presence of lectin and phytoplasmas
DBA	+	mg	−	−	1	1	−	0	0	nd
VVA	+++	mg + fc	+++	mg + fc	1	1	−	1	1	−
PNA	+	mg + fc	+	mg + fc	0	0	nd	1	1	−
RCA	++	fc	++	fc	1	1	−	1	1	−
WGA	+++	mg + fc	+++	mg + fc	2	2	+	1	1	++
LEL	+++	mg + fc	+++	mg + fc	6	6	+++	6	6	+++
GNA	+++	mg + fc	+++	mg + fc	6	6	+++	6	6	+++
LCA	+++	mg + fc	+++	mg + fc	6	6	+++	6	6	+++

*nd* not determined, *fc* filter chamber, *mg* midgut.

### Colonization by FD phytoplasmas of salivary gland cells with glycoproteins interacting with GNA, LCA and LEL lectins

**Figure 3:**
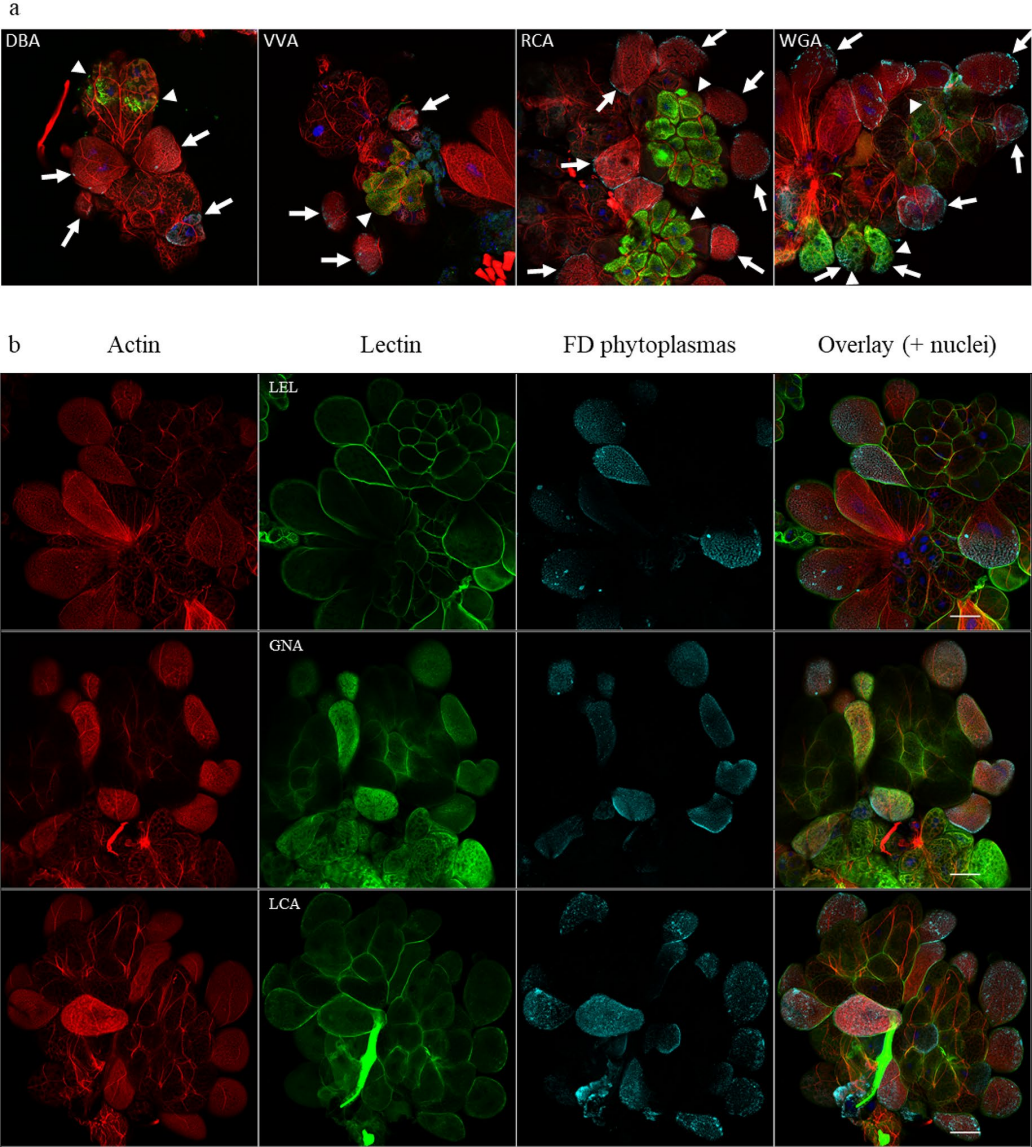
Observation by laser scanning microscopy of fluorescent lectins binding salivary gland carbohydrates of FDP-infected *E*. *variegatu*s. Cell nuclei were stained with DAPI (blue), actin filaments with Alexa 568-phalloidin (red), phytoplasmas with anti-VmpA antibodies and Alexa 633-secondary antibodies (cyan), and FITC-lectins are colored green. (**a**) Overlay pictures of infected salivary gland cells stained with the lectin indicated in each picture. Arrows indicate the FDP-infected cells, and arrowheads indicate the cells recognized by the lectin used. (**b**) View of the infected salivary gland surfaces stained with the lectin indicated in each picture. Scale bar 100 µm.

To correlate FDP colonization and lectin binding, the salivary glands of FDP-infected *E*. *variegatus* were labeled with the same lectins as for the healthy insects and were observed using laser scanning microscopy. In this experiment, no difference was observed between the salivary glands of infected insects and those of noninfected insects (Figs. 2 and 3). No staining was observed with the lectin PNA (Fig. 2 for noninfected insects, and not shown for infected insects). The cells labeled with the lectins DBA, VVA, RCA and WGA (Fig. 3a, arrowhead) were determined not to be infected or to be infected very rarely at low rates for DBA, VVA and RCA and at higher rates for WGA (Fig. 3a, arrow). In contrast, salivary gland cells stained with the fluorescent lectins LEL, GNA and LCA were observed to be infected by phytoplasmas (Fig. 3b). Similar results were obtained when FDP-infected salivary glands of *S*. *titanus* were observed (see Supplementary Fig. S3 online), and these findings are presented in Table 2.

**Figure 4:**
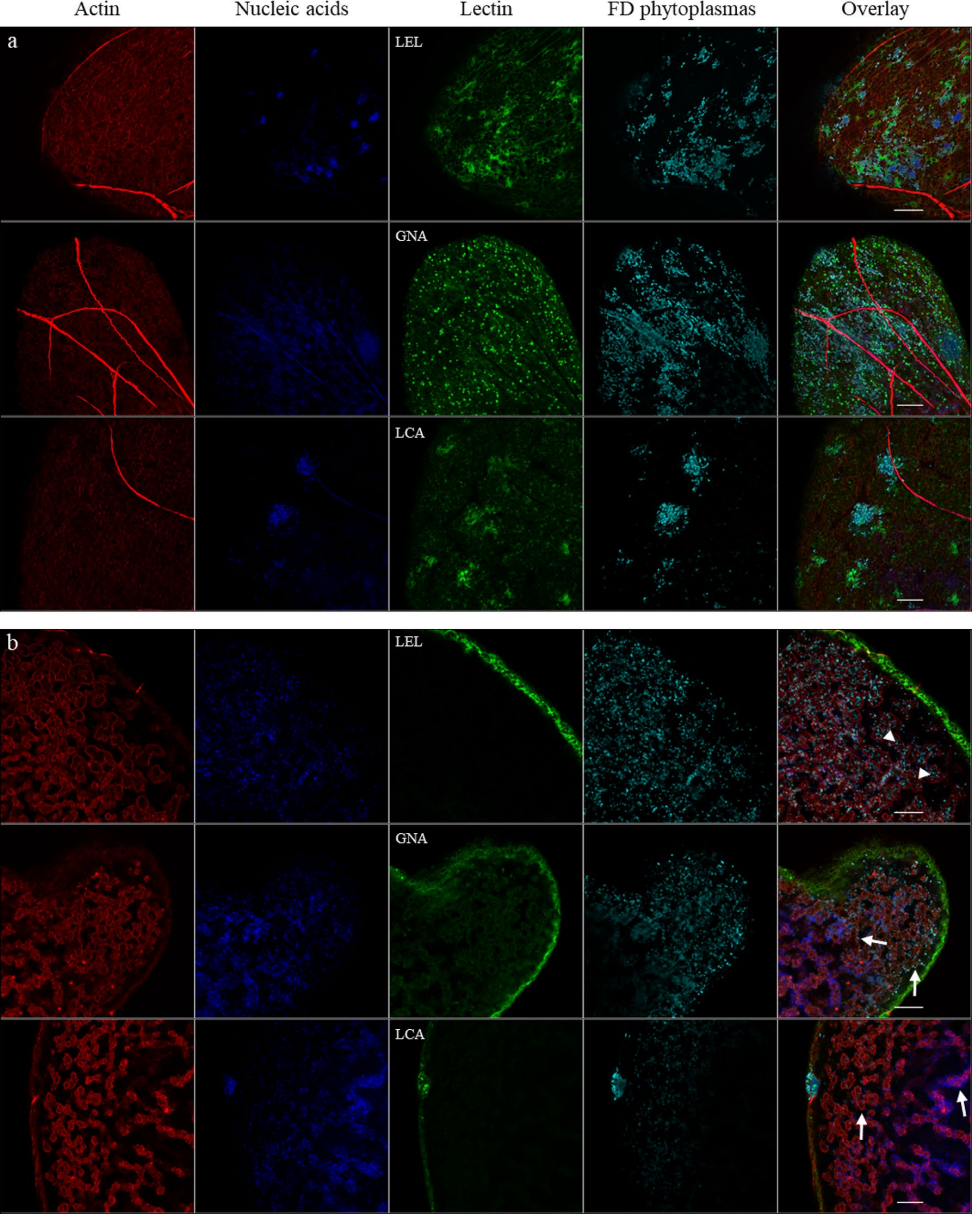
Magnification of salivary glands of FDP-infected *E*. *variegatus* stained with fluorescent LEL, GNA and LCA lectins by laser scanning microscopy with an Airyscan detector. FDPs were stained with anti-VmpA antibodies and Alexa 633-secondary antibodies and (cyan), cell nuclei with DAPI (blue), actin filaments with Alexa 568-phalloidin (red) and glycoconjugates with FITC-lectins LEL, GNA and LCA (green). (**a**) View of the infected salivary gland cell surface. (**b**) View of the inside of infected salivary gland cells. Arrows indicate structures resembling apical membranes described by Koinuma et al.^16^ around which phytoplasmas are grouped. Arrowheads indicate phytoplasmas that crossed the apical plasma membrane. Scale bar 10 µm.

When the salivary glands were observed at high magnification, patches of phytoplasmas could be visualized near carbohydrates bound to GNA, LCA, and LEL at the surface of the cells (Fig. 4a). In the case of the LEL and LCA lectins, dense labeling was observed close to the phytoplasmas. These aggregates were not observed on the salivary gland cells of noninfected insects (Supplementary Fig. S4), suggesting that the adhesion of FDPs at the surface of these cells induced a gathering of glycoproteins recognized by the LEL and LCA lectins. Once the phytoplasmas had crossed the basal membrane, they grouped close to reticulated structures resembling apical membranes, as described by Koinuma et al.^16^. Phytoplasmas were detected around (indicated by arrows in Fig. 4b) and inside (indicated by an arrowhead in Fig. 4b) these intracellular structures, which were labeled through actin staining. In some cells, GNA bound these membranes where phytoplasmas gathered. These structures were not labeled or very poorly labeled with the LEL and LCA lectins.

### Similarity of lectin-labeling patterns between insect vector midgut and salivary gland cells and Euva-6 cells in culture

**Figure 5:**
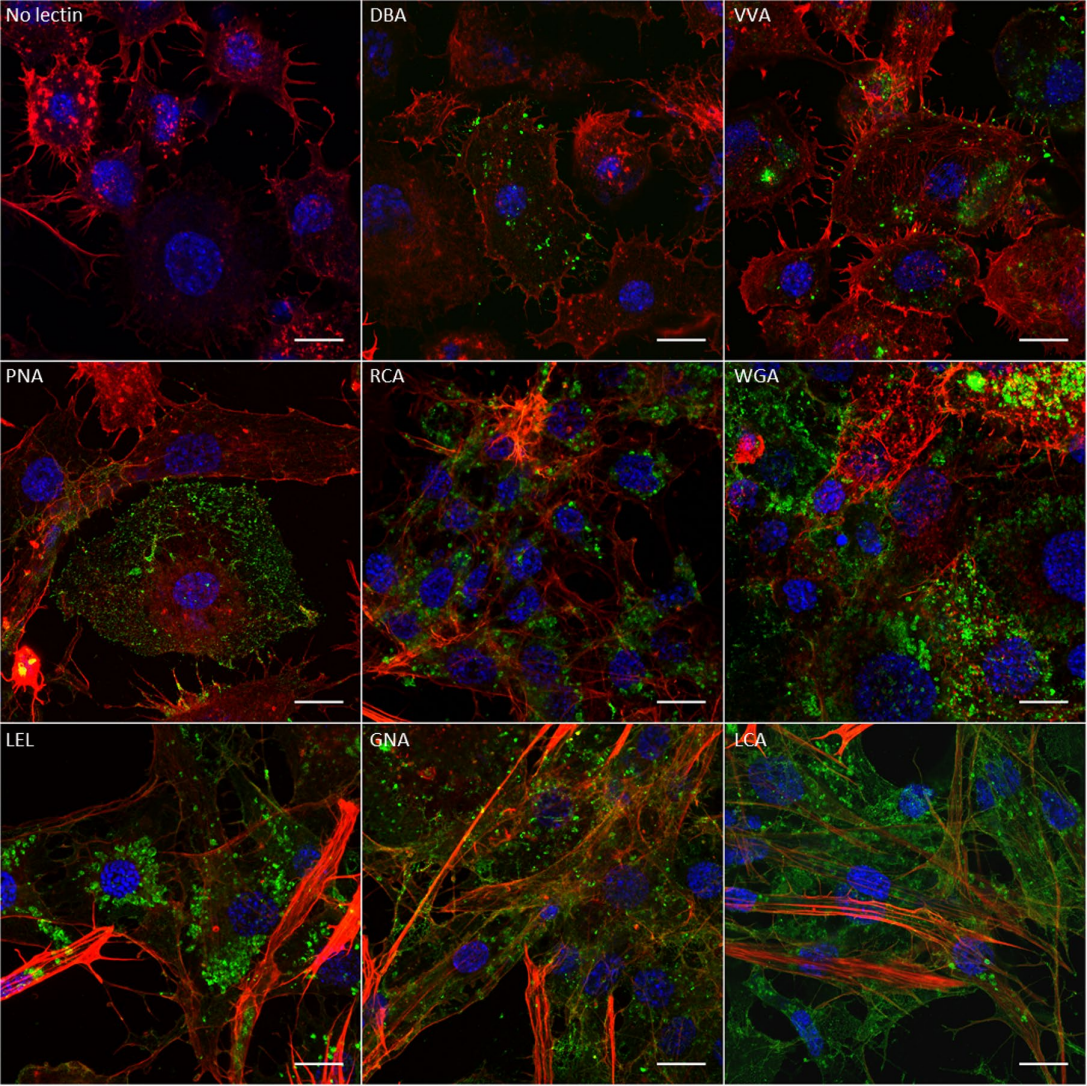
Observation of fluorescent lectins binding Euva-6 cell carbohydrates by laser scanning microscopy with an Airyscan detector. Cell nuclei were stained with DAPI (blue), actin filaments with Alexa 568-phalloidin (red) and glycoconjugates with FITC-lectins (green). The fluorescent lectins used to stain the cells are indicated in each picture except for the control, where fluorescent lectin was omitted. Scale bar 10 µm.

The Euva-6 cell line was established from *E*. *variegatus* embryonated eggs and was primarily composed of three different cell types differentiated by their size and actin filament network. As we previously used Euva cells in culture to elucidate the interactions of VmpA with insect cells, we investigated whether the glycosylation of the Euva-6 cell surface was similar to that of midgut epithelial and salivary gland cells of *E. variegatus* and *S. titanus*. To test this hypothesis, we examined the same lectins as are observed on the salivary glands and midguts of *E. variegatus* and *S. titanus*. The results show that fluorescent lectins bound glycoconjugates present at the surface of Euva-6 cells (Fig. 5). Only a portion of cells possessed carbohydrates recognized by the lectins DBA and PNA. In contrast, stronger and more frequently occurring signals were observed for the lectins VVA, RCA, WGA, LEL, GNA, and LCA, even though the signal was less abundant for the lectin VVA. This result confirms that the Euva-6 cell line is composed of different cell types, as previously shown^33^. Regardless of the lectin observed, the fluorescent lectins were not uniformly distributed on the cells; rather, they were often grouped in patches. These results demonstrated that the lectins VVA, WGA, LEL, GNA, and LCA, which bind the surfaces of midgut and salivary gland cells most widely, also recognize glycoconjugates at the surface of Euva-6 cells in culture. Therefore, we further utilized these Euva-6 cells to test the lectin-like binding capacity of the VmpA protein.

### Modification of VmpA adhesion to *E. variegatus* Euva-6 cells in culture by *N*-acetylglucosamine and mannose

**Figure 6:**
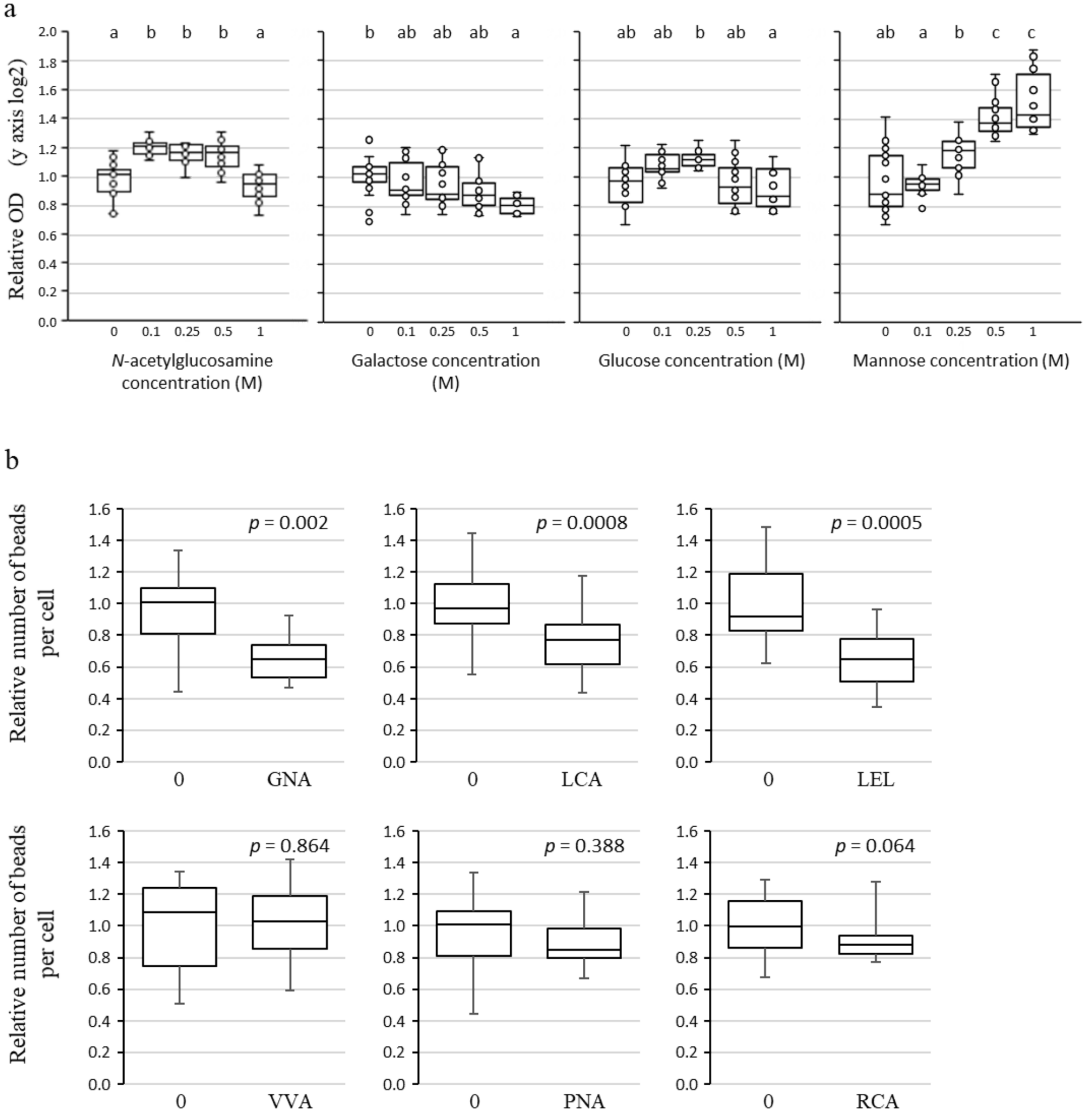
Interaction of VmpA with Euva-6 cells in the presence of sugar and lectins. (**a**) Adhesion of VmpA-His_6_ proteins to Euva-6 cells measured by ELISA in the presence of sugars. The recombinant proteins were first incubated with different concentrations of sugars indicated below the graphs before being added to Euva-6 cells. Boxplots with different letters are significantly different under the ANOVA test of the R commander package of R software version 4.0.3 (R: A language and Environment for statistical computing, R Core Team, R Foundation for Statistical Computing, Vienna, Austria, 2020, https://www.R-project.org). The normal distribution of the value was previously verified using the Shapiro–Wilk normality test of R software (R commander package). (**b**) Adhesion of VmpA-His_6_-coated beads to Euva-6 cells in competition with lectins. Euva-6 cells were preincubated with the lectins indicated above the graphs before being incubated with fluorescent VmpA-His_6_-coated beads. 0 indicates that no lectin was incubated with insect cells. The P values according to the Kruskal–Wallis rank sum test of the software R (R commander package) are indicated above the boxplots.

To determine whether VmpA could interact with carbohydrates, we first performed binding assays in which Euva-6 cells grown on 96-well plates were incubated with VmpA, either with or without prior incubation with sugar inhibitors. The recombinant protein VmpA-His_6_ was preincubated with Gal, Glc, GlcNAc and Man at different concentrations before being transferred to Euva-6 cells. Glc, GLcNAc and Man were chosen because they are specifically recognized by the lectins LCA, LEL and GNA, which were detected close to the phytoplasmas invading the salivary glands. Gal served as control. The OD measured in the control wells, i.e., wells without any sugar added to VmpA, varied between 0.24 and 0.73, depending on the experiment. When GlcNAc and Man were incubated with recombinant VmpA, they modified the fixation of VmpA to Euva-6 cells in a different manner (Fig. 6a). At the smallest concentration used (0.1 M), GlcNAc significantly increased the adhesion of VmpA to insect cells, and decreased adhesion was subsequently observed, which was significant at the 1 M concentration. Man treatment increased the adhesion of VmpA to insect cells in a dose-dependent manner at concentration between 0.1 and 1 M. When VmpA was incubated with Glc, no significant differences were observed, except between the conditions of 0.25 and 1 M concentrations. When VmpA was incubated with Gal, no significant differences were observed, except with the condition of 1 M. These results suggest that VmpA could interact with the sugars GlcNAc and Man and that these interactions modify the adhesion of recombinant VmpA to insect cells.

### Decrease in VmpA-coated bead adhesion to Euva-6 cells in the presence of the lectins GNA, LCA and LEL

Adhesion competition assays were performed to inhibit the adhesion of VmpA-His_6_-coated fluorescent beads to insect cells with lectins. In these experiments, different lectins were preincubated with Euva-6 cells prior to the addition of VmpA-His_6_-coated beads. The number of VmpA-coated beads counted by cell varied from 21 to 29, depending on the experiment. The presence of lectins bound to the cells modified the adhesion of the VmpA-coated beads to Euva-6 cells differently depending on the lectin (Fig. 6b). A significant decrease in adhesion was observed when the cells were preincubated with lectins that bind Man (GNA and LCA) and GlcNAc (LEL). In contrast, when the lectin VVA that binds GalNAc was incubated with Euva-6 cells, no decrease in VmpA-coated beads was observed. A small and not significant decrease was observed when the insect cells were incubated with PNA and RCA lectins that bind Gal and GalNAc, respectively. Taken together, the results suggest that the VmpA protein could bind insect proteins glycosylated with Man and/or GlcNAc residues, similar to the GNA, LCA and LEL lectins.

### Inhibition of interaction between VmpA and insect proteins by GlcNAc and increase by man

**Figure 7:**
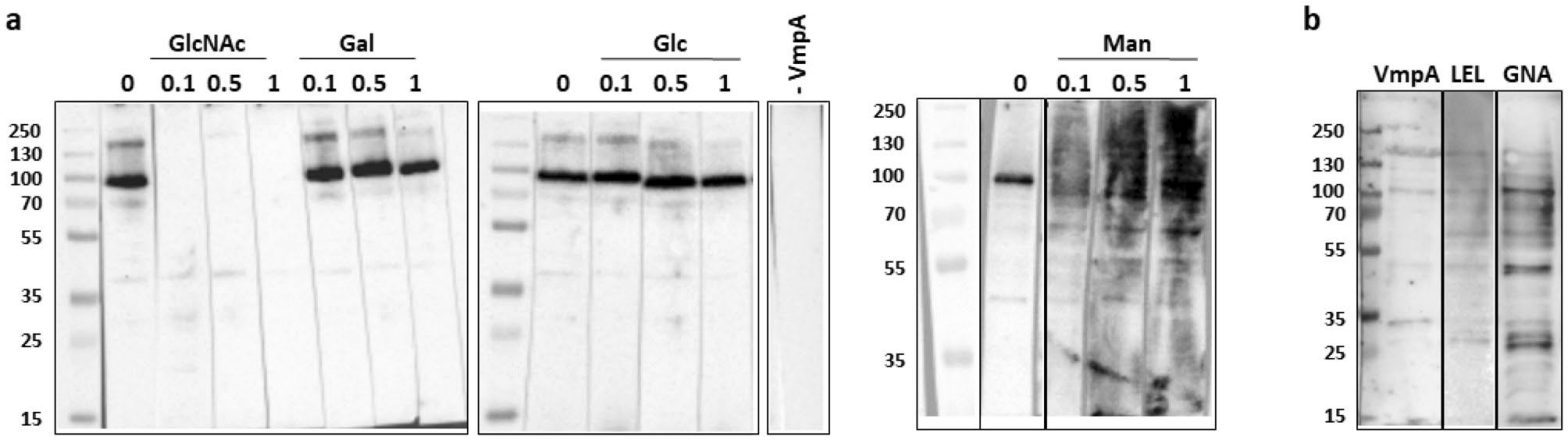
Interaction of VmpA-His_6_ with insect proteins. The results are presented from different blots (full length) framed by black lines. (**a**) Euva-6 proteins from culture passage 7 transferred to nitrocellulose membranes were incubated with recombinant VmpA-His_6_ alone (lane 0) or in the presence of 0.1 to 1 M Gal, Glc, GlcNAc or Man. The presence of VmpA-His_6_ interacting with insect cells was detected using anti-VmpA antibodies. (**b**) Euva-6 proteins from culture passage 32 transferred to nitrocellulose membranes were incubated with recombinant VmpA-His_6_ or the lectins LEL and GNA.

To determine the molecular mass of the proteins that interact with VmpA, protein overlay assays were performed with Euva-6 proteins separated by SDS-PAGE and blotted onto membranes. The results show that VmpA-His_6_ was able to interact essentially with Euva-6 (culture passage 7) proteins with apparent masses of 90–95 kDa (Fig. 7a, column 0). Additional minor signals of interaction were detected for proteins measuring 210, 150, 70, 40 and 28 kDa.

The recombinant protein VmpA-His_6_ was preincubated with GlcNAc, Gal, Glc and Man at various concentrations before incubation with Euva-6 cell proteins. The presence of GlcNAc and Man at increasing concentrations modified the pattern of interaction of VmpA-His_6_ with insect cell proteins, whereas Gal and Glc had no effect (Fig. 7a). When recombinant VmpA-His_6_ was incubated with insect cells in the presence of GlcNAc, only the interaction between VmpA-His_6_ and the protein measuring approximately 40 kDa remained visible. The interaction of VmpA-His_6_ with proteins of greater mass was completely abolished regardless of the concentration of GlcNAc. When VmpA-His_6_ was incubated with Man, interaction with insect proteins was reinforced, mostly for the proteins with high molecular weight. These results are in keeping with previous results showing that in the presence of increased concentrations of Man, recombinant VmpA-His_6_ exhibited stronger adhesion to Euva-6 cells (Fig. 6a). These unexpected results prompted us to examine the effect of Man on VmpA-coated beads without contact with insect cells. Microscopic observations of beads coated with 9 nmol of VmpA-His_6_ and 1 nmol of BSA (standard condition) showed that when the beads had not been incubated with Man, the majority of the spots (36%) contained one to two beads (see Supplementary Fig. S5 online). When the beads had been incubated with Man, the majority of the spots (23%) contained aggregates of 25 to 50 beads per spot, and only 18% of the spots were composed of one to two beads. When the beads were not incubated with Man, only 1% of the spot contained between 51 and 100 beads aggregated (maximum size observed), whereas when the beads were incubated with Man, 13% of the spots contained between 51 and 200 beads and 1% more than 200 beads. Under control conditions, 72% and 62% of beads coated with 1 nmol of BSA contained one to two beads per spot without or with Man incubation, respectively. No more than 51–100 beads per spot were observed with these beads. Thus, these results suggest that once VmpA binds Man, the protein VmpA forms multimers that aggregate VmpA-Hi_6_-coated beads and can increase the affinity of VmpA for the GlcNAc residues.

Taken together, the results showed that VmpA could have lectin-like activity, similar to that of GNA and LEL. Therefore, we compared the profile of the Euva-6 (culture passage 32) proteins that interacted with VmpA with the profiles of the Euva-6 proteins interacting with the LEL and GNA lectins. Even if the two lectins LEL and GNA interacted with many more proteins than VmpA did, the VmpA-interacting proteins had the same molecular weight as the proteins interacting with the LEL and GNA lectins (Fig. 7b). Taken together, these results strongly suggest that VmpA interacts with glycoconjugates containing Man and GlcNAc and binds insect cells similarly to the lectins LEL and GNA.

## Discussion

Circulative transmission of viruses, bacteria and parasites by insects requires crossing of the intestinal tract and salivary glands. For example, when the colonization of salivary gland cells is inhibited, transmission to the plant is decreased or abolished, as demonstrated for phytoplasma and *Spiroplasma citri*^43,44^. The first step of cell colonization is the recognition of surface components by microbe adhesins. Colonization of the digestive tract and salivary glands often requires the recognition of glycoconjugates by lectin-like adhesins. Numerous examples have been described in the literature, and some of them will be detailed below in the discussion. Indeed, in insects, the salivary gland surface and the midgut perimicrovillar membrane are highly glycosylated^37,38^ as was observed in this study for *E*. *variegatus* and *S*. *titanus* (Figs. 1, 2, and see Supplementary Figs. S1 and S2 online), for which a variety of carbohydrates had been detected at the surfaces of these two organs. The most abundant carbohydrates of *E*. *variegatus* and *S*. *titanus* midguts labeled with fluorescent lectins were Man, GalNAc and GlcNAc, as observed for the perimicrovillar membrane of the insect *Triatoma* (*Meccus*) *pallidipennis*, except for GlcNAc^40^. Labeling of carbohydrates at the surfaces of salivary glands, although highly similar, showed slight variations between *E*. *variegatus* and *S*. *titanus*, suggesting some differences in glycoconjugate carbohydrate composition*.* Variations in glycoconjugate carbohydrate composition were previously observed among different arthropods and even among different species of the same genus^36,45,46^. Similar to our case with *E*. *variegatus* and *S*. *titanus,* differences were also demonstrated between the cell types of salivary glands of Anopheles and *Circulifer haematoceps*^38,46^. When phytoplasmas invade salivary glands, they colonize primarily type III cells of *Macrosteles striifrons* in the case of onion yellows phytoplasmas and types III, IV and V of *E*. *variegatus* in the case of FD phytoplasmas^16,47^. We also observed that FDP did not colonize all the different types of *E*. *variegatus* and *S*. *titanus* salivary gland cells. FDP rarely colonized cells containing carbohydrates that link the lectins DBA, VVA and RCA. In contrast, FDP colonized cells recognized by LEL, GNA and LCA lectins. Moreover, LEL, GNA and LCA were the lectins that more extensively bound the apical surface of the midgut. Therefore, it was hypothesized that at least one FDP adhesin may act as a lectin with Man and GlcNAc affinity. The localization in patches of the LEL lectin at the surface of Euva-6 cells resembled the pattern of VmpA-coated bead adhesion to these cells^33^. This finding prompted us to hypothesize that VmpA could be an adhesin with lectin activity.

Our results obtained in vitro with the adhesin VmpA confirmed this hypothesis. Phytoplasmas internalize in the midgut, which is not covered with a chitin tegument, in contrast to the foregut and hindgut. GlcNAc, the carbohydrate monomer composing chitin, was investigated in this study, as it is a core component of N-glycosylation. Chitin degradation products other than GlcNac, such as glucosamine-composed chitobiose and chitotriose, were not examined in our study. Adhesion to Man or GlcNAc was previously documented for several pathogens invading host cells from arthropods to mammals. For example, *Xylella fastidiosa* possesses the afimbrial adhesins HxfA and HxfB and the protein PD1764, which bind GlcNAc residues and chitin of on the foregut cuticle of its insect vectors^41,42^. Inhibition assays with GlcNAc, (GlcNAc)2 or (GlcNAc)3 and lectins with affinity for GlcNAc showed that attachment of *Xylella* to GlcNAc is required for the transmission of the bacterium by its vector^48^. In the case of *Trypanosoma,* diverse lectin-like adhesins appear to be involved in the attachment of the parasite to both midgut and salivary gland barriers of their insect vectors. Incubation of *Trypanosoma cruzi* with Man and, to a lesser extent, with GlcNAc and Gal decreased the adhesion of *T*. *cruzi* to the PMM of *Rhodnius prolixus*^49^. For *Trypanosoma rangeli*, inhibition assays with GlcNAc decreased the attachment of parasites to the salivary glands of *R*. *prolixus* but not Man^35^. Similar interactions were found in mammals, where enteric carbohydrates also function as receptors for bacteria. For instance, the adhesin FimH of *Escherichia coli* type 1 fimbriae binds mannose^50^ and, as it is highly expressed on the bacterial surface, the FimH-mannose interaction enables a large part of the bacterial surface to adhere to epithelial cells^51^.

We showed that adding Man as a potential inhibitor surprisingly increased the adhesion of VmpA to Euva-6 cells and Euva-6 proteins, contrary to GlcNAc, which inhibited the interaction of VmpA with insect proteins. The adhesion of Man to VmpA may induce a change in VmpA conformation, which could result in oligomerization of the adhesin. Indeed, when we mixed VmpA-His_6_-coated beads with Man, we observed aggregation of the beads (see Supplementary Fig. S5 online). One could argue that the phenomenon of VmpA oligomerization could be responsible for the increased detection of VmpA using ELISA tests (Fig. 6); however, this oligomerization could also induce a cooperative effect on the binding of VmpA to GlcNAc. Oligomerization is a common trait of lectins^52–54^. Moreover, autoaggregation of bacterial adhesins has been observed previously. For example, the adhesins TibA and AIDA-I are able to mediate adhesion of enterotoxigenic *E*. *coli* to epithelial cells and autoaggregation of the bacteria^55,56^. The autoaggregation of VmpA following their binding to the surface of salivary gland cells could explain the colony-like structures of FD phytoplasmas observed during the invasion process.

In conclusion, the presence of carbohydrates Man and GlcNAc at the surfaces of the midgut and salivary glands and the sensitivity of VmpA binding to Man and GlcNAc support the hypothesis that insect glycans may serve as recognition sites for the adhesin VmpA. The identification of the insect protein(s) interacting with VmpA may confirm this hypothesis and help to characterize the specificity of FD phytoplasma transmission by vectoring leafhoppers. Recent ecological and genetic surveys have correlated variations in VmpA sequences to the ability of phytoplasma vectotypes to be transmitted by vectors of different leafhopper subfamilies^24^. However, preliminary data suggest that VmpA sequence variations do not fully explain the lack of transmission of FDP vectotype I by *S. titanus.* Variations on the insect side at the level of the VmpA target should be investigated. As glycoconjugate carbohydrate composition was sometimes found to be different between diverse species in the same genus, it could be hypothesized that there is a correlation between the carbohydrate composition of insect vector glycoproteins binding VmpA and the ability of these leafhoppers to transmit FDPs. Identifying leafhopper glycoproteins binding VmpA could also facilitate the development of novel strategies to block FDP transmission.

## Methods

### Insect rearing, phytoplasma strain and Euva-6 cell culture

Healthy *Euscelidius variegatus* leafhoppers were reared in cages on fava beans (*Vicia faba* var. aquadulce) and oats (*Avena sativa*) at 25 °C. Healthy *Scaphoideus titanus* hatched from eggs laid on 2-year-old *Vitis vinifera* canes collected in FD-free vineyards in Burgundy. After the canes were stored at 4–8 °C, they were incubated in boxes at 22 °C with daily water spraying. The L1 larvae were transferred to cages on healthy *V*. *vinifera*.

The phytoplasma strain FD92 (FDP) was originally transmitted to broad bean by infected *S*. *titanus* sampled on FD-diseased vineyards in southwest France^27,57^ and was continuously maintained in broad beans by *E*. *variegatus* transmission, as described by Caudwell et al.^32^.

To obtain infected *E*. *variegatus* and *S*. *titanus*, L4-5 larvae were transferred by groups of 100 on FDP-infected broad bean for phytoplasma acquisition. One week later, *E*. *variegatus* were placed in cages on healthy broad bean and *S*. *titanus* on *V*. *vinifera* for a latency period of 3–4 weeks before use.

The Euva-6 cell line was established from embryos of *E*. *variegatus*, as previously described for Ciha-1 cells^58^. In brief, the eggs were sterilized with bleach solution and then with 70% ethanol. After rinsing, the eggs were ground in modified culture medium made of 350 mL Schneider’s Drosophila medium (Invitrogen), 100 mL Grace’s insect cell culture medium (Invitrogen), 50 mL heat-inactivated fetal bovine serum (Eurobio) and 2 mL G-5 supplement (Invitrogen). The Euva-6 cells were cultivated at 25 °C. After the first colonies developed and the cell line was established, the cells were passed using trypsinization every week with an additional change in the medium during the week.

### Labeling of midguts, salivary glands and Euva-6 cells with lectins

Lectins used in this study were labeled with fluorescein isothiocyanate or were not labeled (Vector Laboratories) (Table 1).

For midgut labeling with fluorescent lectins, healthy *E*. *variegatus* and *S*. *titanus* adults were fed through a parafilm membrane, as described in^33^, with a solution consisting of HEPES 8 mM, sucrose 280 mM, pH 7 plus FITC-lectins (diluted 1:100) for two days. Midguts were dissected after the insects were anaesthetized with CO_2_. The midguts were fixed in 4% paraformaldehyde (Electron Microscopy Science) containing 1% Triton X-100 overnight at 4 °C and then washed 3 times in PBS. The midguts were incubated for 1.5 h with Alexa 568-phalloidin (Invitrogen, diluted 1:40) to stain the actin filaments, and after three washes for 5 min, midguts were incubated with DAPI (1 µg/mL, Sigma) in water to stain nuclei. The organs were mounted with anti-fading ProLong Gold Reagent (Thermo Fisher Scientific), and immunofluorescent samples were imaged using a Zeiss LSM 880 confocal laser-scanning microscope with an objective with a numerical aperture of 0.45. The excitation wavelengths and emission filters were 405 nm/415–487 nm, 488 nm/499–553 nm, 561 nm/579–624 nm and 633 nm/643–695 nm for DAPI, FITC, Alexa 568 and 633, respectively. Fluorochromes were detected sequentially frame by frame. For each experiment, 10–15 organs were observed per condition, and two independent assays were performed.

For salivary gland labeling, healthy and FDP-infected insects were first dissected after the insects were anaesthetized. Salivary glands were fixed in 4% paraformaldehyde containing 1% Triton X-100 overnight at 4 °C and then washed 3 times in PBS. Next, the salivary glands were incubated for 1 h with rabbit anti-VmpA polyclonal antibodies (diluted 1:5000, produced by covalab) and FITC-lectins (diluted 1:100). After three washes, salivary glands were incubated for 1.5 h with Alexa 568-phalloidin (Invitrogen, diluted 1:40) and secondary Alexa 633-conjugated goat anti-rabbit IgG (Thermo Fisher Scientific) at a 1:200 dilution and, after washing, for 5 min in water with DAPI (Sigma). The salivary glands were mounted and observed as previously described for the midguts with objectives with numerical apertures of 0.45 and 1.4. The resolution was improved with an Airyscan detector when the objective with a numerical aperture of 1.4 was used. Excitation wavelengths, which are sequentially individually switched on, are 405 nm, 488 nm, 561 nm and 633 nm for DAPI, FITC, Alexa 568 and 633, respectively. The same emission filter (BP 420–460 nm + LP 500 nm) was used for the 4 channels. For each experiment, 10–15 organs were observed per condition, and two independent assays were performed.

Euva-6 cells at culture passage number 27 were cultivated on cover slips in 24-well plates (Falcon) for 24 h and then fixed in 4% paraformaldehyde for 15 min. The glycoconjugates, actin filaments and nuclei were stained as described above with fluorescent lectins, Alexa 568-phalloidin and DAPI. The cells were mounted and observed as previously described using an Airyscan detector.

### Inhibition and competitive adhesion assays

The VmpA-His_6_ proteins were obtained as previously described^33^. In brief, the proteins were expressed in *Escherichia coli* BL21 Star (DE3) cells containing the plasmid pET28-VmpA-His_6_ and purified on a His-select nickel affinity gel packed column (Sigma) according to the manufacturer’s protocol. For inhibition adhesion assays with sugar, Euva-6 cells of culture passages 7 to 37 depending on the assay were cultivated in 96-well plates (Falcon) and when at confluence, *i.e.,* entirely cover the bottom of the wells, the cells were fixed in 4% paraformaldehyde for 15 min and washed twice in PBS. Recombinant VmpA-His_6_ (20 µg/mL) was preincubated with D-α-mannose (Man), D-α-glucose (Glc) or N-acetylglucosamine (GlcNAc) (Sigma) at different concentrations (1 M, 0.5 M, 0.25 M and 0.1 M) in 0.5 × washing buffer (PBS + 0.05% Tween 20) for 30 min at room temperature (RT). Mixes of 1 µg of VmpA-His_6_ and sugars were then incubated with Euva-6 cells for 1.5 h. The presence of VmpA attached to Euva-6 cells was revealed by a first incubation with rabbit anti-VmpA polyclonal antibodies (1:5000, Covalab) for 2 h, a second incubation with goat anti-rabbit IgG-PAL (1:1000, Sigma) for 1 h, and a third incubation with the substrate pNPP (Sigma) for 2 h at 37 °C. Optical density (OD) was measured at 405 nm using an Epoch-Microplate Spectrophotometer (Biotech Instrument). Five to six independent repetitions (corresponding to different wells) were performed by assay, and three independent assays were grouped to establish the graphs of Fig. 6. The value 1 of the relative OD corresponds to the mean OD measured when only VmpA was added to the Euva-6 cells (control condition, corresponding to condition 0 on the graphs of Fig. 6). Relative OD values were calculated by dividing the OD measured in each different condition by the mean OD of the control condition.

For competitive adhesion assays, Euva-6 cells of culture passages 7 to 37, depending on the assay, were cultivated on coverslips in 24-well plates (Falcon), as previously described (Duret et al*.*, 2014). The red fluorescent and amine-modified beads (4 × 10^9^ beads at 1 µm, Sigma) were covalently coated with 9 nmol of recombinant VmpA-His_6_ proteins plus 1 nmol BSA according to the supplier’s instructions. In this case, the N-terminus of VmpA is predicted to be exposed to the surface of the beads, as is the case in FD phytoplasmas. The coated beads were maintained at 4 °C for one or two days before use. The coating of fluorescent beads was verified using immunofluorescence observations and measuring the remaining uncoated proteins as previously described^33^. Lectins used for competitive adhesion assays were GNA, LCA, LEL, VVA, PNA and RCA. Euva-6 cells were incubated with 500 µL of lectins (20 µg/mL) in Schneider’s Drosophila medium for 1 h at 25 °C. After two washes in Schneider’s Drosophila medium, 2 × 10^6^ VmpA-His_6_-coated beads were added for 1 h at 25 °C. After three washes, the cells were fixed with 4% paraformaldehyde, and the cell nuclei were stained with DAPI (Sigma) for 5 min. The samples were mounted in the antifading ProLong Gold reagent and analyzed with a fluorescence microscope (Nikon Eclipse E800) with an objective with a numerical aperture of 0.5. DAPI and fluorescent beads were observed with the following excitation and emission filters: 340–380 nm/435–485 nm and 510–560 nm/wavelengths longer than 590 nm. Each experiment was repeated three times independently. For each experiment, 20 to 25 fields, with approximately 300 cells per field, were observed randomly. The counting of beads per cell was performed with the free software package ImageJ (http://imagej.nih.gov/ij/). The relative numbers of adherent beads per cell in the different conditions were calculated as above, and the mean of the bead numbers under the control condition (without lectin indicated by 0 in Fig. 7) corresponded to a value of 1.

To observe the effect of mannose on the aggregation of VmpA-his_6_-coated beads, red fluorescent and amine-modified beads (4 × 10^9^ beads at 1 µm, Sigma) were covalently coated with 9 nmol of recombinant VmpA-His_6_ proteins plus 1 nmol BSA or with 1 nmol of BSA as a control according to the supplier’s instructions. The beads were incubated with or without 1 M Man in 0.5 X washing buffer (PBS + 0.05% Tween 20) for 30 min at room temperature (RT). Beads were subsequently spotted on glass, mounted in the antifading ProLong Gold reagent and analyzed with a Zeiss Axio Imager fluorescence microscope with an objective with a numerical aperture of 0.5. Bead fluorescence was detected with the following excitation/emission bandpass filter: 542–582 nm/wavelength longer than 593 nm. The counting of beads was performed with the free software package ImageJ.

### Overlay assays

Proteins of Euva-6 cells of culture passage 7 or 37 were prepared by crushing the insect cells in Rx buffer^18^. Euva-6 proteins (12.5 µg) were then separated by 10% SDS-PAGE and electrotransferred to nitrocellulose membranes for 1.5 h. Membranes were incubated in PBS containing 5% nonfat dry milk for 4 h.

For overlay assays with recombinant VmpA-His_6_, the membranes were subsequently incubated overnight with 50 µg of recombinant VmpA-His_6_ at 4 °C in PBS. After washing three times for 10 min in PBS + 0.2% Tween 20 and twice for 5 min in PBS (repeated after each incubation), membranes were incubated with anti-VmpA antibodies (1:5000) for 1 h at room temperature and then with HRP-conjugated goat anti-rabbit (1:20,000, Sigma) for 1 h in PBS + 2% nonfat dry milk. The signal was detected with ChemiDoc (Bio-Rad) after the addition of the chemiluminescent substrate SuperSignal West PICO (Thermo Fisher Scientific).

For inhibition overlay assays, different concentrations of sugar (0.1, 0.5 and 1 M) were preincubated with VmpA-His_6_ for 30 min before overnight incubation of VmpA-His_6_ plus sugar with Euva-6 proteins.

For overlay assays with LEL and GNA, saturated membranes were incubated overnight with the biotinylated lectins LEL or GNA (5 µg/mL, Vectors Laboratories) for 30 min. After three washes in PBS 0.05% Tween 20, the presence of biotinylated lectins was revealed using the VECTASTAIN Elite ABC-HRP kit (Vector laboratories) and the chemiluminescent substrate SuperSignal West PICO according to the manufacturer's instructions.

## Supplementary Information

Supplementary Information.
