# Supplementary material for: Interactions between the flavescence dorée phytoplasma and its insect vector indicate lectin-type adhesion mediated by the adhesin VmpA
Source: Sci Rep. 2021 May 27;11:11222. doi: 10.1038/s41598-021-90809-z (PMC8160148; doi:10.1038/s41598-021-90809-z)
Supplement: Supplementary file 1 — Supplementary Information. [file 41598_2021_90809_MOESM1_ESM.pdf]

Interactions between the flavescence dorée phytoplasma and its insect vector indicate lectin-type adhesion mediated by the adhesin VmpA

Nathalie Arricau-Bouvery[1], Sybille Duret[1], Marie-Pierre Dubrana[1], Delphine Desqué[1], Sandrine Eveillard[1], Lysiane Brocard[2], Sylvie Malembic-Maher[1] and Xavier Foissac[1]

[1]Univ. Bordeaux, INRAE, Biologie du Fruit et Pathologie, UMR 1332, F-33140 Villenave d'Ornon, France
[2]Univ. Bordeaux, CNRS, INSERM, Bordeaux Imaging Center, BIC, UMS 3420, US 4, F-33140 Villenave d'Ornon, France

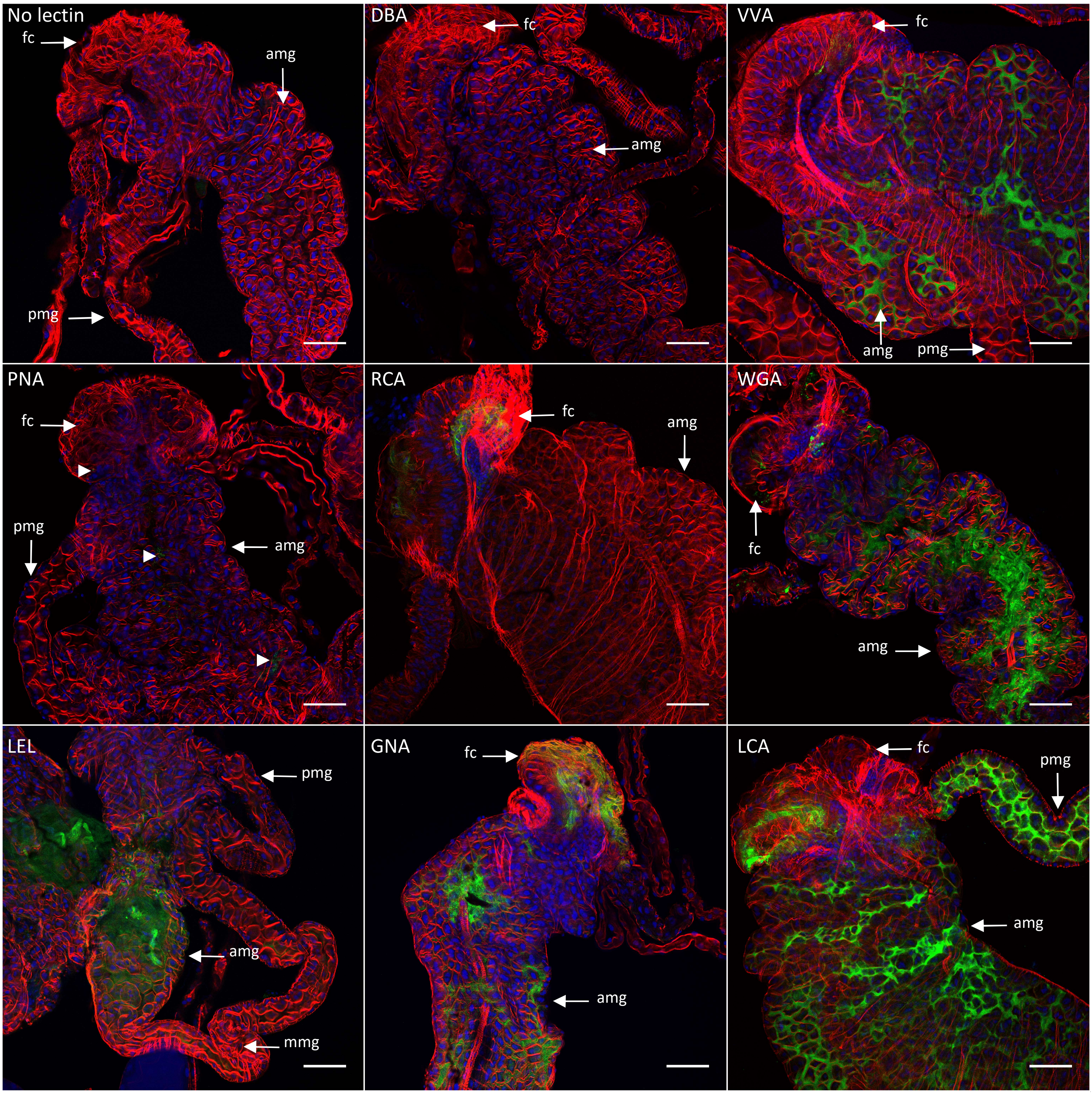

Figure S1. Observation of ingested fluorescent lectins binding midgut carbohydrates of *S. titanus* by laser scanning microscopy. Cell nuclei were stained with DAPI (blue) and actin filaments with Alexa 568-phalloidin (red). The FITC-lectins were colored in green. The fluorescent lectins used to stain the cells are indicated in each picture except for the control where fluorescent lectin was omitted. amg, anterior midgut; mmg, middle midgut; pmg, posterior midgut; fc, filter chamber. Arrowheads in the panel PNA indicate the presence of fluorescence. Scale bar 100 µm.

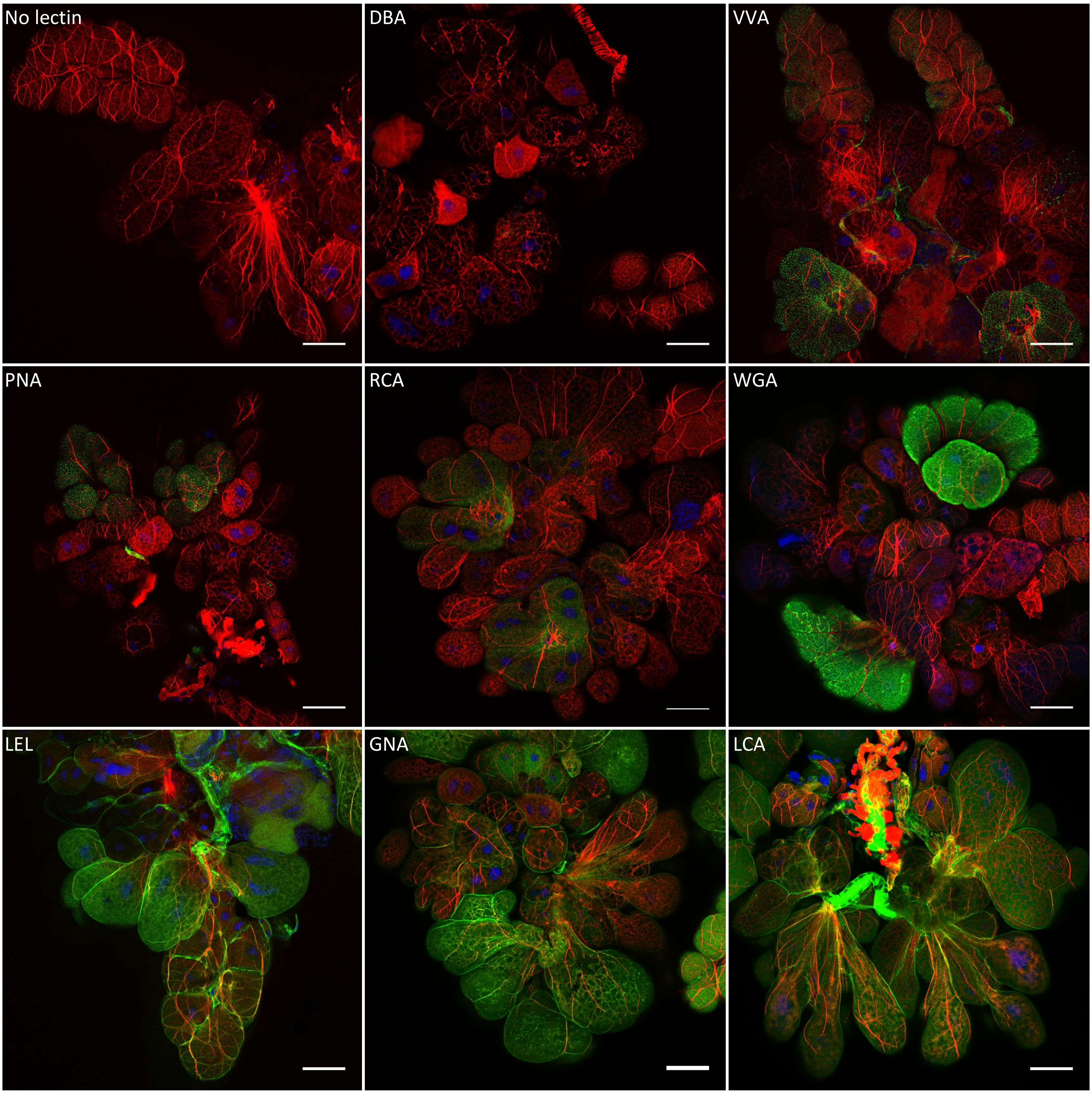

Figure S2. Observation of fluorescent lectins binding salivary gland carbohydrates of *S. titanus* by laser scanning microscopy. Cell nuclei were stained with DAPI (blue), actin filaments with Alexa 568-phalloidin (red). The FITC-lectins were colored in green. The fluorescent lectins used to stain the cells are indicated in each picture except for the control where fluorescent lectin was ommited. Scale bar 100 µm.

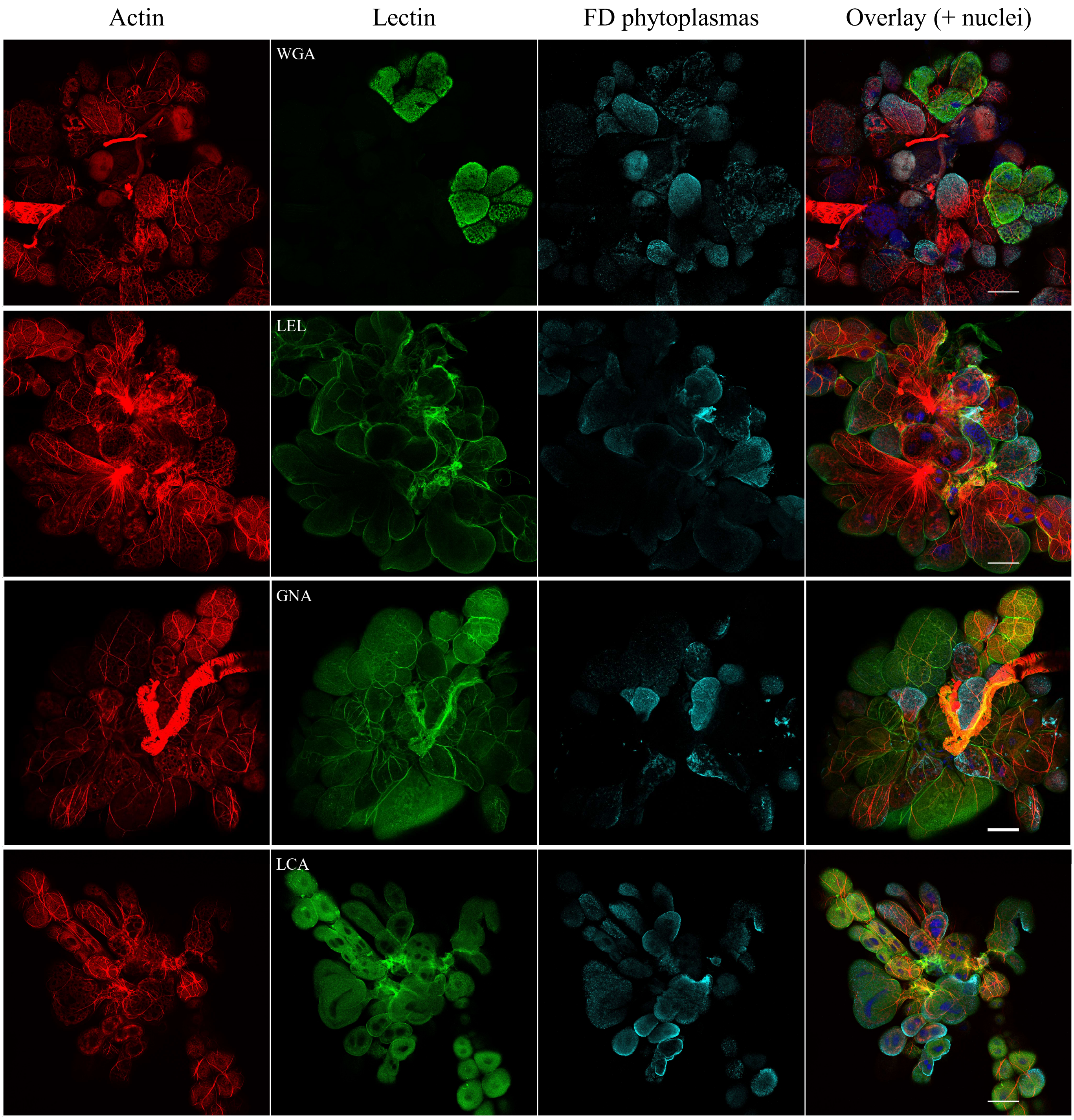

Figure S3. Observation of salivary glands of FDP infected *S. titanus* stained with fluorescent lectins by laser scanning microscopy. FDP were stained with anti-VmpA antibodies and Alexa 633-secondary antibodies (cyan), cell nuclei with DAPI (blue), actin filaments with Alexa 568-phalloidin (red) and glycoconjugates with FITC-lectins (green). Scale bar 100 µm.

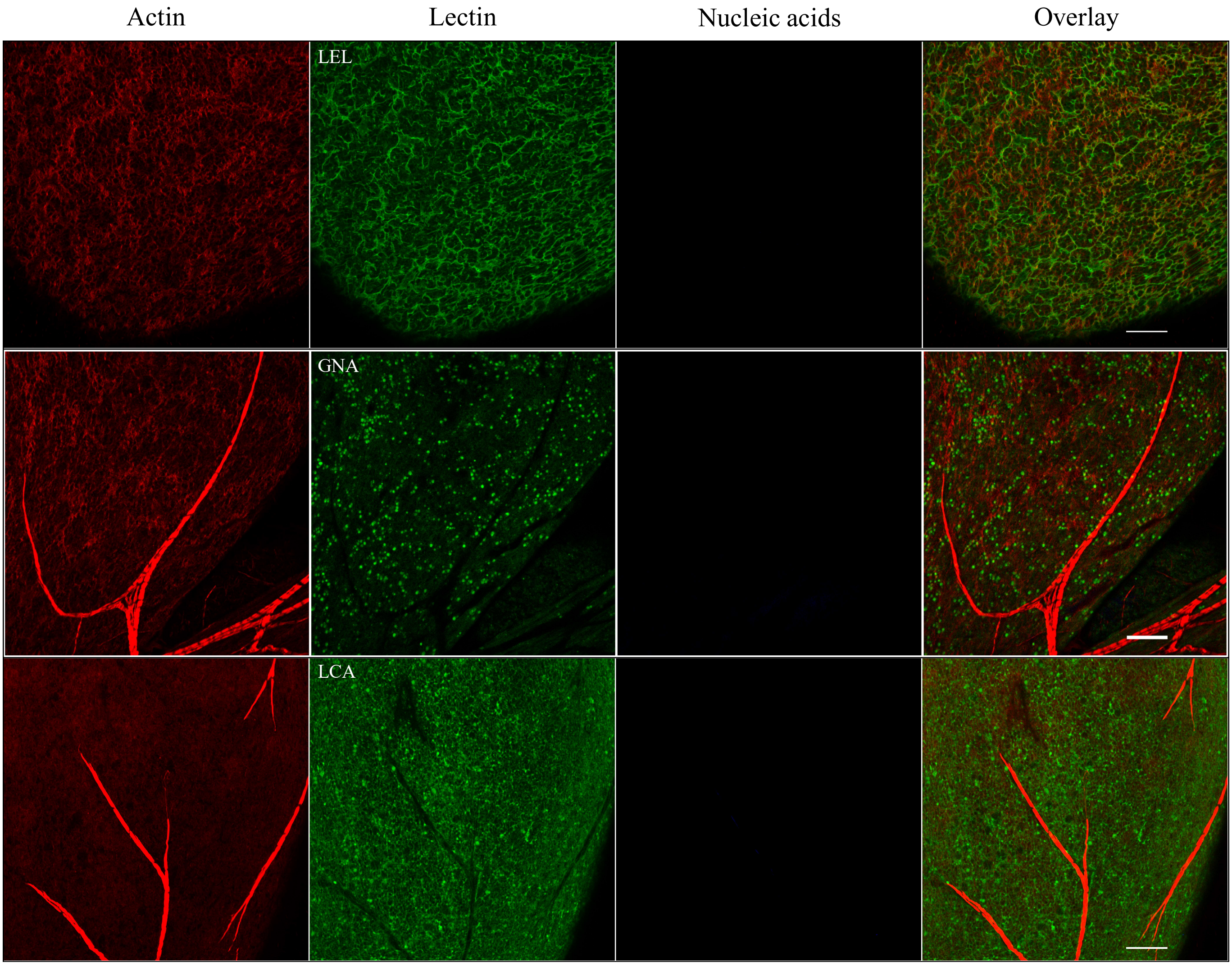

Figure S4. Observation of the surface of salivary glands of noninfected *E. variegatus* stained with fluorescent lectins by laser scanning microscopy with an Airyscan detector. Actin filaments were stained with Alexa 568-phalloidin (red), glycoconjugates with FITC-lectins (green) and cell nuclei with DAPI (blue). Scale bar 10 µm.

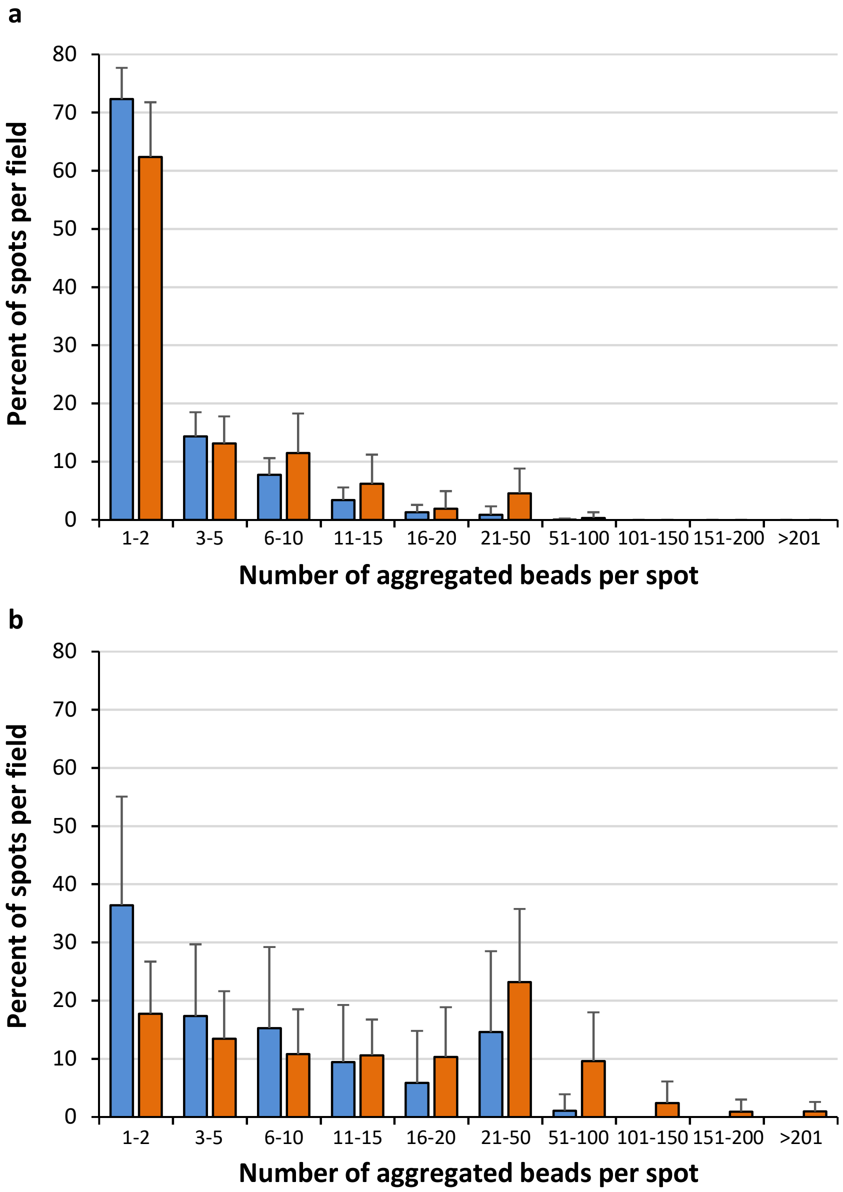

Figure S5. Aggregation of VmpA-His$_6$-coated beads in presence of mannose. The beads coated with 1 nmole of BSA (a), and the beads coated with 1 nmole of BSA plus 9 nmole of VmpA-His$_6$ (b) were incubated without (blue) or with 1M of Man (orange) for 30 min before to be placed between slide and coverslip. After observation with epifluorescence microscope, the images were analyzed using Image J software. The bar plots represent the mean of percentage of spot number corresponding of spots containing aggregated beads indicated under the graph.
